# Evolution of the class C GPCR Venus flytrap modules involved positive selected functional divergence

**DOI:** 10.1186/1471-2148-9-67

**Published:** 2009-03-27

**Authors:** Jianhua Cao, Siluo Huang, Ji Qian, Jinlin Huang, Li Jin, Zhixi Su, Ji Yang, Jianfeng Liu

**Affiliations:** 1 Key laboratory of Molecular Biophysics of the Ministry of Education, College of Life Science and Technology, Huazhong University of Science and Technology, Wuhan, Hubei, PR China; 2 State Key Laboratory of Genetic Engineering, Institute of Genetics, School of Life Sciences, Fudan University, Shanghai, PR China; 3 Institutes of Biomedical Sciences, Fudan University, Shanghai, PR China; 4 Center for Evolutionary Biology, Fudan University, Shanghai, PR China

## Abstract

**Background:**

Class C G protein-coupled receptors (GPCRs) represent a distinct group of the GPCR family, which structurally possess a characteristically distinct extracellular domain inclusive of the Venus flytrap module (VFTM). The VFTMs of the class C GPCRs is responsible for ligand recognition and binding, and share sequence similarity with bacterial periplasmic amino acid binding proteins (PBPs). An extensive phylogenetic investigation of the VFTMs was conducted by analyzing for functional divergence and testing for positive selection for five typical groups of the class C GPCRs. The altered selective constraints were determined to identify the sites that had undergone functional divergence via positive selection. In order to structurally demonstrate the pattern changes during the evolutionary process, three-dimensional (3D) structures of the GPCR VFTMs were modelled and reconstructed from ancestral VFTMs.

**Results:**

Our results show that the altered selective constraints in the VFTMs of class C GPCRs are statistically significant. This implies that functional divergence played a key role in characterizing the functions of the VFTMs after gene duplication events. Meanwhile, positive selection is involved in the evolutionary process and drove the functional divergence of the VFTMs. Our results also reveal that three continuous duplication events occurred in order to shape the evolutionary topology of class C GPCRs. The five groups of the class C GPCRs have essentially different sites involved in functional divergence, which would have shaped the specific structures and functions of the VFTMs.

**Conclusion:**

Taken together, our results show that functional divergence involved positive selection and is partially responsible for the evolutionary patterns of the class C GPCR VFTMs. The sites involved in functional divergence will provide more clues and candidates for further research on structural-function relationships of these modules as well as shedding light on the activation mechanism of the class C GPCRs.

## Background

**Figure 1:**
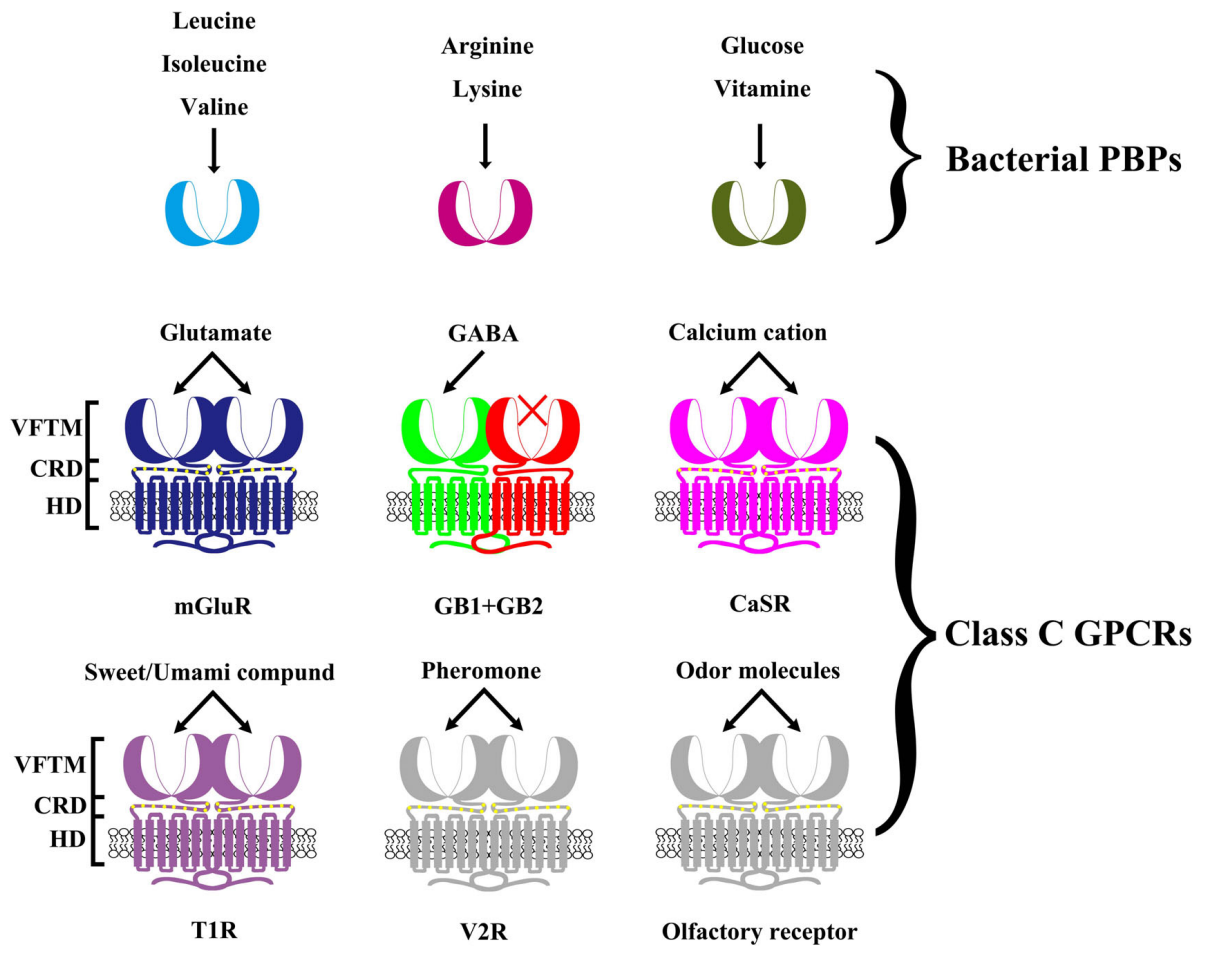
**Schematic representation of class C GPCRs**. Class C GPCRs share structural similarity with bacterial PBPs but have higher ligand-binding specificity. These receptors have a common structure consisting of an N-terminal extracellular VFTM, a transmembrane HD connected with VFTM via CRD, and a variable C-terminal intracellular tail. The bacterial PBPs have extensively ligand-binding properties, which are involved in transporting small nutritional molecules such as amino acids and vitamins. The class C GPCRs have a physiological function in shaping ligand-binding specificity. The GB2 group lacks the ability to directly bind ligands.

The G protein-coupled receptors (GPCRs) are seven transmembrane receptors coupled to G proteins and represent a major group of cell-surface receptors that constitute 3.5% of the genome in vertebrates [1]. These receptors play a major role in intercellular communication and act as receptors for most hormones and neurotransmitters. The GPCRs are involved in the perception of the environment, being activated by taste compounds, pheromones, odorants and even photons [2]. Several classes of the GPCRs have been defined based on sequence similarity [1,3,4]. The class C GPCRs are mainly composed of metabotropic glutamate receptors (mGluRs), gamma-aminobutyric acid type B receptors (GABA_B_Rs), Ca^2+^-sensing receptors (CaSR), taste receptors (T1R), pheromone receptors (V2R) and olfactory receptors[1,2]. They play a key role in the physiology of various types of epilepsy as well as in nociception and drug addiction [5]. These receptors structurally possess an extracellular Venus flytrap module (VFTM) where agonists bind and a heptahelical transmembrane domain (HD) which is responsible for G protein activation [6-11]. For most receptors, with the exception of GABA_B_R, the cysteine-rich domain (CRD) can act as a molecular link between the VFTD and HD domains. Moreover, the VFTMs share structural similarity with bacterial periplasmic amino acid-binding proteins (PBPs), such as the leucine-binding protein (LBP) and the leucine/isoleucine/valine-binding protein (LIVBP) [7,8,12-14]. Interestingly, the bacterial PBPs show extensive ligand-binding properties and can transport small nutritional molecules such as amino acids and vitamins [15,16]. In contrast, the class C GPCR VFTM's physiological function is involved in shaping ligand-binding specificity and GB2 has even lost the ability to bind ligands (Figure 1). The class C GPCRs can form homodimers whereas GABA_B_R is a heterodimer and is composed of two homologous subunits, GABA_B1 _(GB1) and GABA_B2 _(GB2) [17-21].

Although ligand-binding ability are a typical character of the class C GPCR VFTMs, different receptors have different ligand-binding specificities. Moreover, the GB2 receptors have lost the ability to bind ligands but have maintained their function to can activate G proteins [22]. The overall structural similarity between GPCRs and bacterial PBPs imply that there is a common origin among class C GPCRs via internal domain duplication. As a result, the VFTMs provide an interesting evolutionary case to investigate gene duplication and functional divergence events.

Unlike the bacterial PBPs which can bind various different ligands, most class C GPCRs expressed in the central nerve system can bind only one kind of natural ligand, implying that the VFTMs of class C GPCRs have undergone partial loss of function such as the ability to bind different ligands as well as gaining other unknown functions. Meanwhile, it would be interesting to know whether functional alterations in the VFTMs were the result of extensive changes in selective constraints (different evolutionary rate) at those sites involved.

In the present study we undertook an extensive phylogenetic analysis for the VFTMs of five typical groups of class C GPCRs. By inspecting the amino-acid sites, we report that altered selective constraints derived from positive selection resulted in the functional divergence in the VFTM domains of class C GPCRs (and this occurred after three continuous gene duplications). Our study provides a new insight into understanding the ligand-binding specificity and how the activation or modulation mechanism is refined in the class C GPCRs.

## Results

### Phylogeny inference of the VFTMs

**Figure 2:**
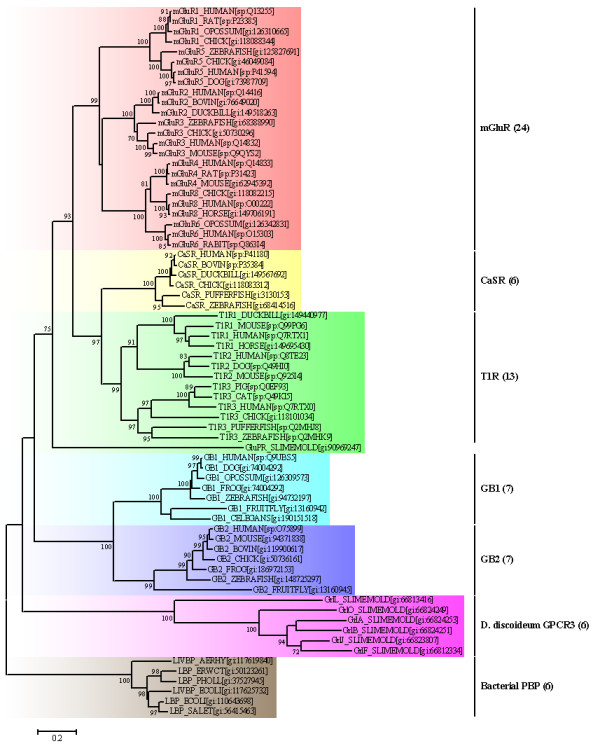
**The bootstrap tree was inferred by the NJ method with JTT model and gamma-distributed rates**. Homologous bacterial PBPs were used as an outgroup to root the trees. Bootstrap values of more than 70% were presented on the node. The accession numbers and corresponding database names (gi for GenBank, sp for Swiss-Prot) for each sequence are placed in square brackets. The total numbers for each class are put in parenthesis followed by the class name.

A phylogenetic analysis of the aligned protein sequences showed that the VFTMs of class C GPCRs fell into three major classes: the mGluR class, a second sensing receptor class consisting of the CaSR and the T1R, and a third less related class consisting of two homologous subunits of the GABA_B_R, GB1 and GB2. A distinct group of bacterial PBPs was used to root the tree dendrogram (Figure 2). In order to validate the tree, the ML dendrogram was inferred based from the reduced protein sequences. The results show that the NJ tree and the ML tree had similarly identical phylogenetic topology (see additional file 1). The monophyletic topology of the VFTMs suggests that there is a common originating ancestor for the class C GPCRs. The average sequence identity between GB1/GB2 VFTMs with bacterial PBPs is lower (7.5% and 8.5%, respectively), however, it's medium (30.6%) between GB1 and GB2. These phenomena implied that the VFTMs of GABA_B_R being related to the bacterial PBPs and were distant homologous group of the class C GPCRs.

### Type I functional divergence of the VFTMs

In order to determine shifted selective constraints in the VFTMs of class C GPCRs, the coefficients of functional divergence (θ) were calculated and based on pairwise sequence comparisons (Table 1). The results show that all θ values were significantly greater than zero (*p *< 0.05), supporting the hypothesis of altered selective constraints had occurred in the VFTMs of class C GPCRs. Moreover, the functional branch lengths (*b*_*F*_) of each VFTM group were estimated according to the functional distance matrix, arbitrarily constraining *b*_*F*_(PBP) = 0 was used to root the scale. The result statistically rejected the null hypothesis of equal functional branch lengths (*p *< 0.05), which implies that the functional role of the VFTMs was different to their ancestral role. The rejection result statistically provides evidence for functional divergence in the VFTMs of class C GPCRs. In particular, *b*_*F*_(GB2) produced the highest value suggesting that GB2 maintained a larger shifted evolutionary rate since the duplication events.

**Table 1:** Coefficients of functional divergence (θ) for all pairwise comparisons of the VFTMs.

	**PBP**	**mGluR**	**CaSR**	**T1R**	**GB1**	**GB2**
**PBP**	0	0.730 ± 0.076	0.761 ± 0.124	0.698 ± 0.091	0.833 ± 0.139	0.637 ± 0.100
**mGluR**	1.311	0.446	0.338 ± 0.106	0.446 ± 0.055	0.787 ± 0.109	0.737 ± 0.101
**CaSR**	1.43	0.413	0.459	0.001 ± 0.022	0.650 ± 0.141	0.906 ± 0.132
**T1R**	1.196	0.591	0.001	0.648	0.826 ± 0.147	0.915 ± 0.127
**GB1**	1.789	1.549	1.049	1.751	0.802	0.384 ± 0.123
**GB2**	1.013	1.334	2.36	2.467	0.485	1.061

The upper-right triangle shows the functional divergence (θ) values for all pairwise comparisons, data is presented as value ± standard error, the lower-left triangle shows the functional distance between two groups, the diagonal shows the functional branch length (*b*_*F*_) values of each group, arbitrarily constraining *b*_*F*_(PBP) = 0.

The three continuous duplication events showed varying effects on shaping the site-specific altered selective constrains of the class C GPCR VFTMs. The result of functional divergence on each duplication event show that the first and second duplication events significantly induced site-specific altered selective constraints to generate the GABA_B_R and mGluR groups, but the third duplication separating the sensing-receptors and the homologous subunits of GABA_B_R group failed to significantly change the site-specific shift of evolutionary rate (Table 2).

**Table 2:** Likelihood ratio test (LRT) of functional divergence for each duplicate event of the VFTMs.

Group 1	Group 2	Duplicate event	θ ± S.E.(ML)	LRT	*P*
mGluR	GB1	I	0.787 ± 0.109	52.076	<0.01
mGluR	GB2	I	0.737 ± 0.101	53.163	<0.01
CaSR	GB1	I	0.650 ± 0.141	21.342	<0.01
CaSR	GB2	I	0.906 ± 0.132	47.410	<0.01
T1R	GB1	I	0.826 ± 0.147	31.777	<0.01
T1R	GB2	I	0.915 ± 0.127	51.798	<0.01
mGluR	CaSR	II	0.338 ± 0.106	10.241	<0.01
mGluR	T1R	II	0.446 ± 0.055	66.438	<0.01
CaSR	T1R	III	0.001 ± 0.022	0.292	NS
GB1	GB2	III	0.384 ± 0.123	5.686	NS

I, II and III represent the first, second and third duplication events, ML and S.E. stand for maximum likelihood and standard error, respectively. The *p *values are the critical value of χ^2 ^distribution with df = 1 (degree of freedom). NS means not significant at 1% level.

**Figure 3:**
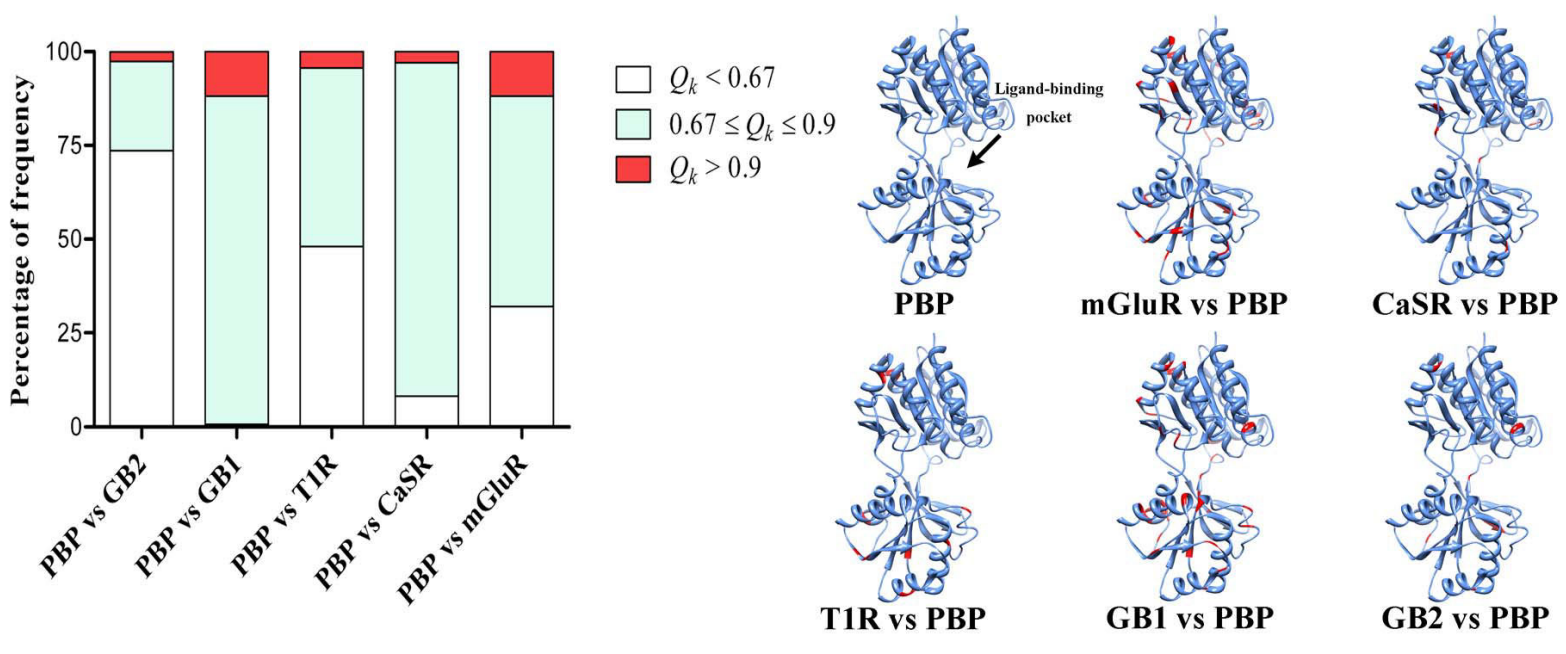
**The percentage distribution of posterior probability (*Q*_*k*_) and the localization of sites with *Q*_*k *_> 0.9**. 269 sites were investigated in bacterial PBPs and the class C GPCR VFTM domains. A bacterial PBP crystal structure [PDB: 2LIV] was used as the template to model the 3D structures. The arrow represents the ligand-binding pocket region. Sites with *Q*_*k *_> 0.9 are depicted in red.

For refining the sites involved in altered functional constrains in the VFTMs, the site-specific profile included 269 sites based on posterior probability (*Q*_*k*_) was used to identify the critical sites responsible for the functional divergence. The percentage distributions of *Q*_*k *_frequencies showed that mass altered functional constraints existed between the class C GPCR VFTM domains and bacterial PBPs (Figure 3). The *ad hoc *high probability components (*Q*_*k *_> 0.9) corresponding to the sites with a high probability of contribution to functional divergence were variable between different pairwise comparisons. By comparing mGluR and GB1 with bacterial PBPs, 32 sites (11.9%) were detected, while CaSR, T1R and GB2 had 8 sites (3.0%), 12 sites (4.5%) and 7 sites (2.6%) respectively. Interestingly, comparisons within the class C GPCRs showed that GB2 showed significant change in altered functional constraints, but little difference was observed between the GB1 and GB2 groups (see additional file 2). By comparing with bacterial PBPs, the sites involved in altered functional constraints with *Q*_*k *_> 0.9 were conserved in specific groups and generally determined the structural characteristics of the VFTMs. These sites were extremely variable for each different group but all were located around the ligand-binding pocket (Figure 3). Moreover, there was little variation within a group and variability with bacterial PBPs. Accordingly, all 7 sites with *Q*_*k *_> 0.9 in GB2 were excluded under this criterion. These phenomena imply that GB1 and GB2 possessed dramatically different characteristics for altered functional constraints, with GB1 showing similarity with class C GPCRs, and GB2 showing similarity for bacterial PBPs.

These results suggest that these sites probably played an important role in defining ligand-binding specificity for the VFTMs in class C GPCRs. In contrast, radical amino acid substitutions with very different chemical properties were found at the same positions in bacterial PBPs, thus indicating that altered selective constraints is related to the functional divergence between bacterial PBPs and the VFTMs in class C GPCRs.

**Figure 4:**
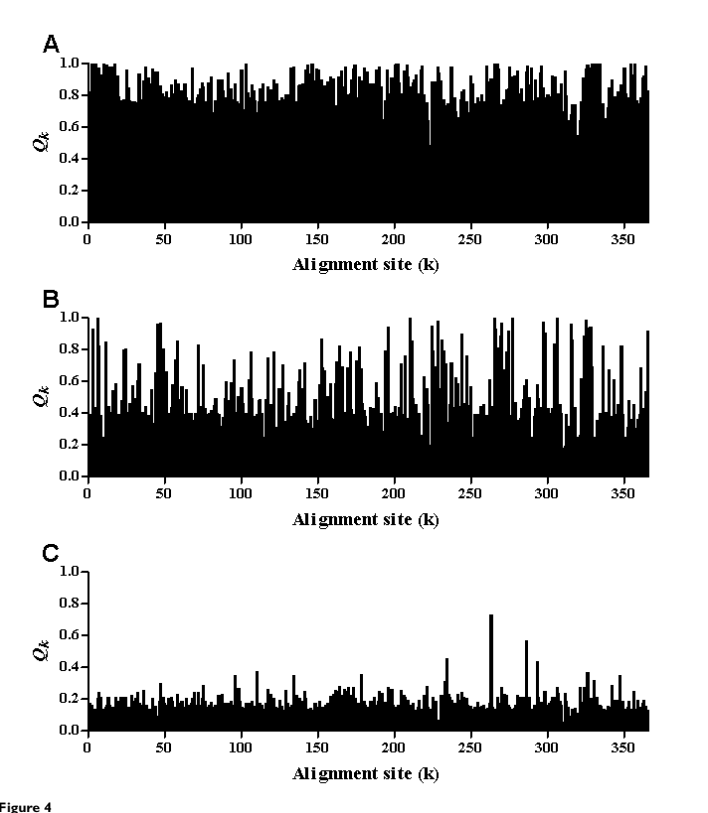
**Identification of sites that show functional divergence related status after duplication events**. A large portion of sites with high posterior probabilities (*Q*_*k*_) had undergone shifted rates after the first duplication event (A), however, only a small portion had shifted rates after the second duplication event (B), while the third duplication event had no effect on shifted rates (C).

The site-specific profiles were used to identify the sites that had functionally diverged after duplication events. The results showed that the majority of sites had undergone shifted rates after the first duplication event, as indicated by the high posterior probabilities. However, a small proportion of sites had undergone shifted rates after the second duplication event, while after the third duplication event there was no effect on shifted rates (Figure 4). These results imply that the first and second duplicate events were mainly responsible for functional divergence and characterizing the different groups, while the third duplicate separated different members within a group as exemplified by the GABA_B_R group and sensing receptors group.

### Positive selection in the VFTM-coding DNA sequences

The nonsynonymous/synonymous substitution rate ratio (ω = *d*_*N*_/*d*_*S*_) of the VFTMs in class C GPCRs, measures the selective pressure exerted at the protein level and thus indicates its evolutionary characteristics, was conducted by pairwise comparison of the human and mouse VFTM-coding sequences. The LRT comparison for variability in the selective pressure among VFTM sites showed large variability in selective pressure amongst the VFTM sites (Table 3). With the selective pressure variation among VFTM sites established, positive selection was tested by two model comparisons. In each comparison, the null model does not allow the presence of sites under positive selection, while the alternative model allows for selection. The test statistics of both comparisons showed high significance and resulted in the rejection of the null models. This indicates that the presence of these sites evolved under positive selection in the VFTMs. Note that type I functional divergence may be detected under diversifying selection or a loss of selective constraints, which is known to be an important process affecting many GPCRs, these functional sites evolved positive selection may be not totally driven by positive selection, however, the model comparisons confirmed that positive selection contributed the functional divergence without doubt.

**Table 3:** LRT statistics 2Δℓ = 2(ℓ_1 _– ℓ_0_) for model comparisons.

Alternative model vs. Null model	2Δℓ	df	χ^2^_1%_	*p*
M2 (positive selection) vs. M0 (one-ratio)	20.551	3	11.345	< 0.01
M2 (positive selection) vs. M1 (nearly neutral)	15.834	2	9.210	< 0.01

χ^2^_1% _values are the critical value of χ^2 ^distribution at 1% level with appropriate degree of freedom.

### Molecular time scale estimation of the VFTMs

**Figure 5:**
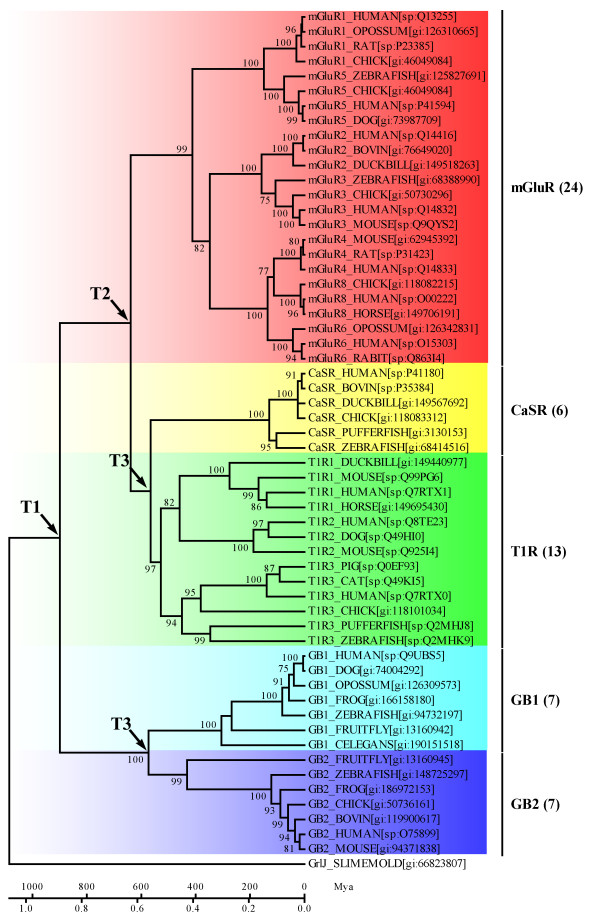
**The linearized NJ tree was used to convert evolutionary distances to the relative molecular time scale**. A GABA_B_-like receptor from *Dictyostelium discoideum *(slime mold), GrlJ, was used as an outgroup to root the tree. Bootstrap values of more than 70% are presented on the node. T_1_, T_2 _and T_3 _indicate time points of the first, second and third gene duplication events respectively. According to the global clock, we estimate T_1 _= 899 Mya, T_2 _= 638 Mya and T_3 _= 565–573 Mya. The split time of *Dictyostelium discoideum *(1085 Mya) was used to calibrate the timescale.

The linearized NJ tree of the orthologous VFTMs under the global clock model suggests that three continuous duplication events had occurred in early stages of vertebrates. Based on the calibration of GrlJ in *Dictyostelium discoideum*, the first duplication event occurred at 899 Mya (T1) resulting in the split of the GABA_B_R group and other class C GPCRs. The mGluR group resulted from the second duplication, occurred at 638 Mya (T2). The third duplication estimated at 573-565 Mya (T3) gave rise to a split resulting in the sensing-receptors (CaSR and T1R) and the GABA_B_R subunits (GB1 and GB2) (Figure 5). Note that many tissue-specific gene families follow similar patterns, raising the possibility of a large-scale duplication in early vertebrates [23,24]. Because of differential selection pressures on different duplicate genes or groups, the time estimation in terms of the molecular clock was taken as an approximation.

### Reconstruction of the ancestral VFTMs

**Figure 6:**
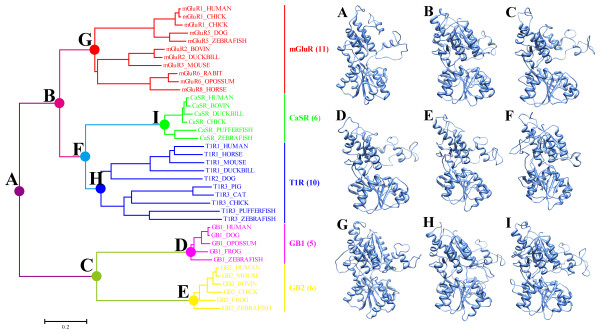
**Reconstruction of the ancestral VFTMs from class C GPCRs**. Nine extinct ancestral amino acid sequences were inferred from 38 aligned present-day sequences and NJ tree topologies. The three-dimensional (3D) structures of ancestors were modelled on the structure of rat VFTM mGluR1 [PDB: 1EWK].

Nine extinct ancestral amino acid sequences were derived from on 38 aligned present-day sequences and the NJ tree topologies. To examine how sequence changes in the VFTMs during the evolutionary process might affect the chemical properties and the ligand-binding pockets, the ancestral three-dimensional (3D) structures were homologous modelled on rat VFTM mGluR1 (Figure 6). The results show that some of the sequence changes in the VFTMs during evolution could cause substantial alterations between the interfaces of the ligand-binding pockets. Considering the structural changes within the VFTMs and the different structural variation between groups together, the correlation between structure and function of the VFTMs suggests that molecular adaption was a result of functional divergence. These radical changes could affect the binding between specific ligands and the class C GPCRs.

## Discussion

Gene or domain duplications have long been thought to be the primary driving events for producing evolutionary novel genes. In our study we have shown that gene duplication plays an important role in the evolutionary process of class C GPCRs. After undergoing three continuous duplications, the GB2 group retained their original structure and functions and are similarly related to bacterial PBPs except for their ligand-binding abilities. In contrast, the other class C GPCR groups under relaxed evolutionary constraints and functional divergence, lead to the diversification and formation of the mGluR, CaSR, T1R and GB1 groups. Because of freely accumulating amino acid replacements, type I functional divergence events resulted in shaping the unique characteristics of each group within class C GPCRs. When sites at the periphery of the ligand-binding pockets were replaced by different amino-acids there was no serious deleterious effects on the survival, the rudiments of each group were fundamentally shaped. In contrast, the well-conserved sites in the ligand-binding pockets are highly variable, which may result in diversification and different ligand specification. In particular, the VFTMs of GB1 and CaSR groups appear to have acquired novel functions for binding new types of ligands, thus explaining the dramatic difference in amino acid composition. Meanwhile, the GB2 group may have functionally diverged through gene duplication events, which would lead to the acquisition of unknown or loss of intrinsic functions.

Although the VFTMs of class C GPCRs were distant originated from bacterial PBPs, their basic ligand-binding ability was intrinsically identical. However, the role was changed from transporting nutrient substances in bacteria to initiating signal transduction in class C GPCRs. The functional alterations under the positive selected functional divergence evolved the complexity of the VFTMs of class C GPCRs.

According to Darwinian theory, complexity derived by a stepwise process of elaboration and optimization under natural selection [25,26]. The VFTMs of the class C GPCRs provides an illustration of this theory. Our results indicate that the functions of VFTMs were generated by molecular exploitation, which recruit of older molecules (PBPs), previously constrained for a different role, into a new functional complex (class C GPCRs). The complexity in the class C GPCR VFTMs consequently arose the biological complexity by a stepwise Darwinian process. In addition, further evidence indicate that positive Darwinian selection played an indispensable role in the origin and evolution of the genes involved in brain development and perception [27]. Our results provide an important insight on how the role of positive selection has a strong effect on the development on the evolutionary process of the class C GPCR VFTM domains.

Finally, the functional divergence rates shift for the class C GPCR HD domains show a similar pattern of stepwise change in amino acid replacement resulting in changes in G-protein coupling ability and different ligand recognition. Despite the lack of experimental evidence for the origination of the HD domains, we observed a group of sites that was highly conserved in class C GPCRs, implying their common origination of seven transmembrane domains. According to the evolutionary pattern of class C GPCRs and the related duplicate events, we propose that an ancestor composed of the bacterial PBP-like domain and a rhodopsin-like transmembrane domain acted as the precursor for the class C GPCRs. The GABA_B_R group arose through a series of point mutations after the first duplicate event. Consequently, two possibilities can result from the selective constraint differences for gene duplication: one being that it may become more conserved in groups such as mGluR, CaSR, T1R or GB1, which induced new functions; the other one may become more variable in groups such as GB2, which resulted in functional relaxation or loss of functions.

## Conclusion

Our results demonstrate that type I functional divergence involved positive selection is partially responsible for driving the evolution of class C GPCRs. Moreover, three internal duplication events had occurred within the class C GPCR VFTMs at the early stage of vertebrates, resulting in the present class C GPCRs. The sites involved in functional divergence may provide extra clues and widen our search for more candidates for further research on the relationship between structures and function, as well as shedding light on the activation mechanism of class C GPCRs.

## Methods

### Data collection

The sequences investigated in this study were obtained from GenBank  and Swiss-Prot  non-redundant databases by manually using gapped BLAST and PSI-BLAST search tools [28,29]. The protein tertiary structures were collected from the Protein Data Bank  by using accession number searches and the family pattern data were retrieved from HOVERGEN  and Pfam database . After removing partial and redundant sequences, the final dataset produced 70 complete sequences involving 24 species that included *Drosophila melanogaster*, *Caenorhabditis elegans *and *Dictyostelium discoideum*.

### Multiple alignment and phylogenetic reconstruction

Multiple alignments were conducted by ClustalW program [30] with default parameters, followed by manual editing using BioEdit [31]. The phylogenetic trees were produced by the neighbor-joining (NJ) method [32] with the Jones-Taylor-Thornton (JTT) probability model and gamma-distributed (a = 1.0) rates among the sites were inferred by using MEGA4 software [33]. Bootstrap with 1000 repetitions was carried out to assess the confidence degree of nodes in the phylogenetic trees. The maximum likelihood (ML) method was used for the phylogenetic reconstruction to validate the tree topology. By using Proml program in PHYLIP package [34] with hidden Markov model (HMM) rates, gamma-distributed rates approximated by 5 rate categories, with coefficient of variation of rates = 1.0. For estimation of divergence time, a linearized NJ tree was used to convert the average distance of protein sequences to the molecular time scale under the global clock model [24,35,36]. In this study, a GABA_B_-like receptor from *Dictyostelium discoideum *(slime mold), GrlJ, was used as the root (1085 million years ago, Mya) to calibrate the time scale [37,38].

### Analysis of type I functional divergence

Type I functional divergence analysis was carried out as previously described [39-42] by DIVERGE software [43]. Coefficients of functional divergence (θ), an indicator for the level of type I functional divergence among two homologous gene clusters, were calculated by DIVERGE with null hypothesis θ = 0. The sites (k) with critical contribution to overall functional divergence were predicted according to their posterior probabilities (*Q*_*k*_), an indicator for the level of functional constraints [39]. The sites with *Q*_*k *_> 0.67 were only meaningful for type I functional divergence in the present study. A matrix of type I functional distance (*d*_*F*_), defined as *d*_*F *_= -ln(1 - θ), was created by using all θ values of all pairwise clusters. As the independence assumption, *d*_*F*_(A, B) = *b*_*F*_(A) + *b*_*F*_(B), the functional branch length of a given cluster, *b*_*F*_, was estimated by non-negative least-square method implemented by MATLAB software.

### Homologous molecular modelling

The homologous models of the VFTMs of class C GPCRs were generated using x-ray crystal structures of rat mGluR1 [PDB: 1EWK] and two PBPs from *Escherichia coli *[PDB: 2LIV and 2LBP] as templates. Models were manually refined with ViTO [44] using the sequence alignment of the rat mGluR1 VFTM. Final models were built using Modeler9v3 [45] and evaluated using dynamic evolutionary trace as implemented in ViTO. The figures were prepared using UCSF Chimera software [46].

### Test of positive selection on the VFTMs

In order to estimate positive selection of the VFTMs, three models, M0 (one ratio), M1 (near neutral) and M2 (positive selection), were conducted by the CODEML program implemented in PAML4b package [47] based on the codon of VFTMs-coding sequences. The nonsynonymous/synonymous substitution rate ratio (ω = *d*_*N*_/*d*_*S*_) indicating the difference of selective constraints was also calculated. Assuming that the synonymous substitution is virtually neutral, ω > 1 indicates positive selection, ω < 1 indicates negative selection, and ω ≈ 1 indicates neutral evolution [39]. If the alternative model indicates that an estimated ω > 1 and the likelihood ratio test (LRT) statistic, 2Δℓ = 2(ℓ_1-_ℓ_0_), is greater than the corresponding critical values of the χ^2 ^distribution, then positive selection can be inferred [48].

### Reconstruction of ancestral sequences

The ancestral amino acid sequences were inferred by distance-based Bayesian method implemented by the Ancestor program [49]. The alignment of present-day sequences and the NJ tree topologies were used to estimate each ancestral node based on the branch length and the JTT model of amino acid substitution. The result was evaluated by the average accuracy.

## Authors' contributions

JHC carried out the study, and participated in its design. SLH participated in the design of the study and performed the statistical analysis. JQ, JLH and ZXS participated in its design and helped to draft the manuscript. LJ participated in the coordination and revision of the manuscript. JY participated in its design and coordination. JFL conceived the study, participated in the coordination of the project and helped to draft the manuscript. All authors read and approved the final manuscript.

## Supplementary Material

Additional File 1 **The ML tree has similarly identical phylogenetic topology with the NJ tree.** Homologous bacterial PBPs were used as an outgroup to root the trees. The accession numbers and corresponding database names (gi for GenBank, sp for Swiss-Prot) of each sequence are in square brackets. The total numbers of each class are in parenthesis followed by class name. Click here for file

Additional File 2 **The percentage distribution of sites involved in altered functional constrains within class C GPCRs.** Using class C GPCRs, 269 sites were investigated based on posterior probability (*Q*_*k*_). Compared with other groups, GB2 showed significant changes in altered functional constrains while GB1 did not reveal variable change. T1R and CaSR showed no altered functional constrains as they belong to the same group of sensing receptors in class C GPCRs. Click here for file
