# Supplementary figures and images for: Evolution of the class C GPCR Venus flytrap modules involved positive selected functional divergence
Source: BMC Evol Biol. 2009 Mar 27;9:67. doi: 10.1186/1471-2148-9-67 (PMC2670285; doi:10.1186/1471-2148-9-67)

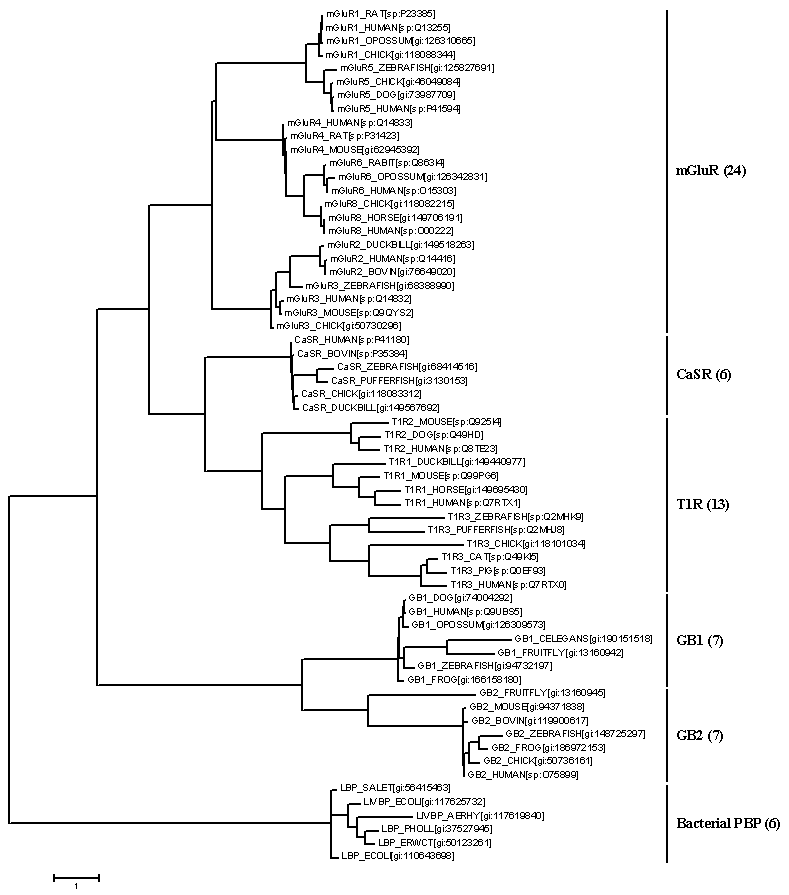

Supplement: Additional File 1 — The ML tree has similarly identical phylogenetic topology with the NJ tree. Homologous bacterial PBPs were used as an outgroup to root the trees. The accession numbers and corresponding database names (gi for GenBank, sp for Swiss-Prot) of each sequence are in square brackets. The total numbers of each class are in parenthesis followed by class name. [file 1471-2148-9-67-S1.png]

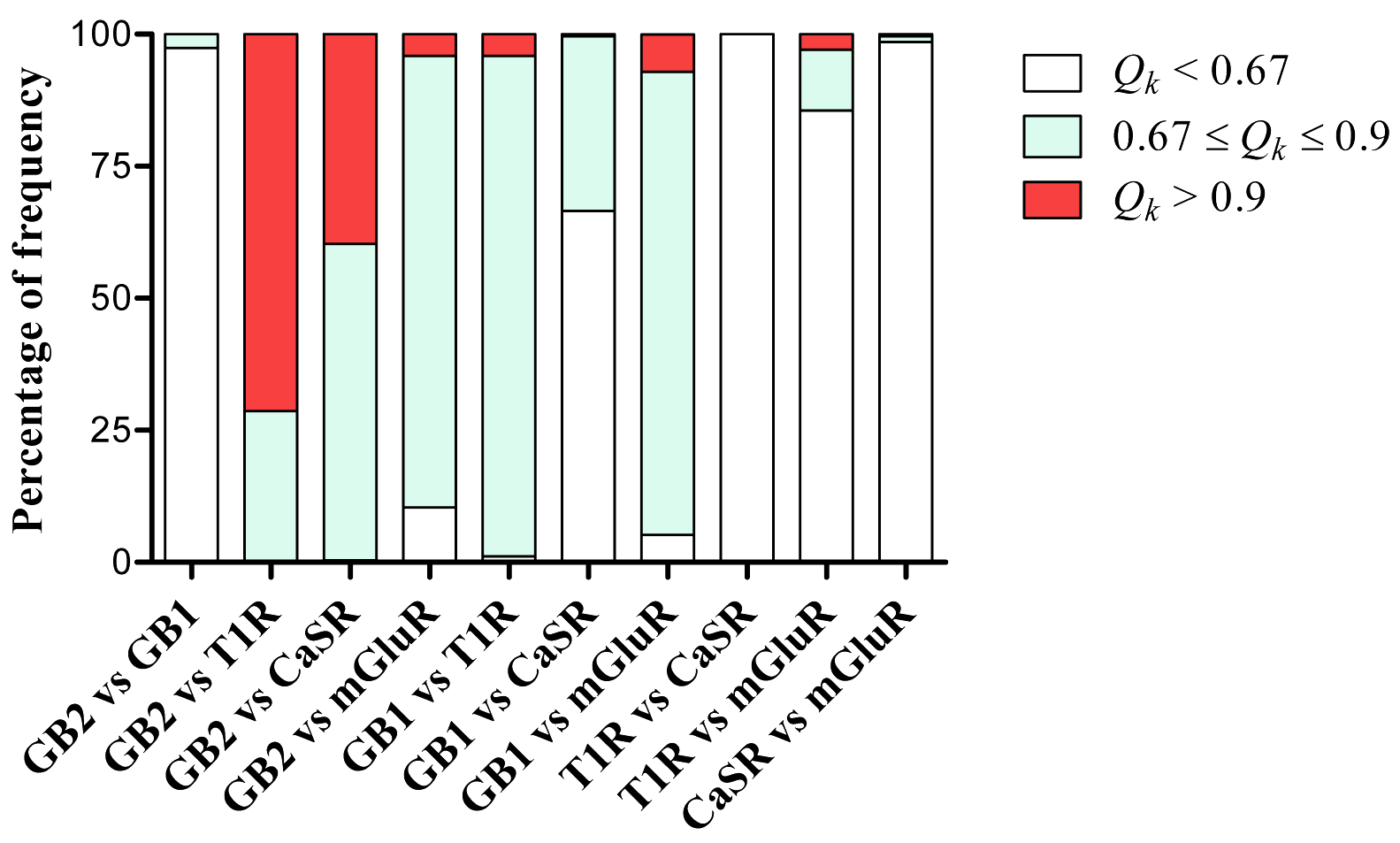

Supplement: Additional File 2 — The percentage distribution of sites involved in altered functional constrains within class C GPCRs. Using class C GPCRs, 269 sites were investigated based on posterior probability (Qk). Compared with other groups, GB2 showed significant changes in altered functional constrains while GB1 did not reveal variable change. T1R and CaSR showed no altered functional constrains as they belong to the same group of sensing receptors in class C GPCRs. [file 1471-2148-9-67-S2.png]
